# During prolonged low-intensity exercise, caffeine alters blood glucose levels

**Published:** 2016

The effects of caffeine versus maltodextrin during exercise were observed in patients with type 2 diabetes. Researchers examined the effects on blood pressure (BP), heart rate (HR) and blood glucose (BG) levels associated with the intake of caffeine in comparison to maltodextrin (CHO) during prolonged periods of low-intensity exercise in patients with type 2 diabetes.

Researchers conducted a pilot study on eight individuals with type 2 diabetes who were aged 55 ± 10 years. The participants either received 1 g/kg of CHO or 1.5 mg/kg of caffeine before undergoing exercise. They then exercised for 40 minutes, executed at 40% HR reserve, and recovered for 10 minutes.

Their BP and exertion, assessed by the Borg scale, were checked every two minute, and their BG levels were checked every 10 minutes. The ANOVA test was used for statistical analysis, and a p-value < 0.05 indicated statistically significant results.

Neither of the treatments produced significant changes in BP and HR. However, 1.5 mg/kg caffeine significantly reduced BG levels by 75 mg/dl (65% CI; p < 0.05) as opposed to 1 g/kg maltodextrin, which produced no significant change in BG levels during the 40-minute period of exercise.
